# Functional Analysis of the Citrate Activator CitO from *Enterococcus faecalis* Implicates a Divalent Metal in Ligand Binding

**DOI:** 10.3389/fmicb.2016.00101

**Published:** 2016-02-09

**Authors:** Víctor S. Blancato, Fernando A. Pagliai, Christian Magni, Claudio F. Gonzalez, Graciela L. Lorca

**Affiliations:** ^1^Laboratorio de Fisiología y Genética de Bacterias Lácticas, Instituto de Biología Molecular de Rosario, Consejo Nacional de Investigaciones Científicas y TécnicasRosario, Argentina; ^2^Department of Microbiology and Cell Science, Genetics Institute, Institute of Food and Agricultural Science, University of FloridaGainesville, FL, USA

**Keywords:** *Enterococcus*, metalloprotein, citrate, FadR family, FCD domain

## Abstract

The regulator of citrate metabolism, CitO, from *Enterococcus faecalis* belongs to the FCD family within the GntR superfamily. In the presence of citrate, CitO binds to *cis*-acting sequences located upstream of the *cit* promoters inducing the expression of genes involved in citrate utilization. The quantification of the molecular binding affinities, performed by isothermal titration calorimetry (ITC), indicated that CitO has a high affinity for citrate (*K*_*D*_ = 1.2 ± 0.2 μM), while it did not recognize other metabolic intermediates. Based on a structural model of CitO where a putative small molecule and a metal binding site were identified, it was hypothesized that the metal ion is required for citrate binding. In agreement with this model, citrate binding to CitO sharply decreased when the protein was incubated with EDTA. This effect was reverted by the addition of Ni^2+^, and Zn^2+^ to a lesser extent. Structure-based site-directed mutagenesis was conducted and it was found that changes to alanine in residues Arg97 and His191 resulted in decreased binding affinities for citrate, as determined by EMSA and ITC. Further assays using *lacZ* fusions confirmed that these residues in CitO are involved in sensing citrate *in vivo*. These results indicate that the molecular modifications induced by a ligand and a metal binding in the C-terminal domain of CitO are required for optimal DNA binding activity, and consequently, transcriptional activation.

## Introduction

Members of the GntR superfamily of transcription factors, Pfam PF00392 (Bateman et al., 2002), are found in diverse bacterial genomes. This group of proteins was first described in 1991 and named after the gluconate operon repressor in *Bacillus subtilis* (Haydon and Guest, 1991). GntR-like regulators are known to control many fundamental cellular processes, such as motility (Jaques and Mccarter, 2006), development (Hoskisson et al., 2006), antibiotic production (Ostash et al., 2011), antibiotic resistance (Truong-Bolduc and Hooper, 2007), plasmid transfer (Reuther et al., 2006), and virulence (Casali et al., 2006). However, most of the specific small molecules that act as a signal to regulate the expression of the respective genes are unknown.

The canonical scaffold of the GntR superfamily consist of a DNA-binding winged helix–turn–helix topology (HTH motif, followed by a β-hairpin) in the N-terminus of the protein. The effector-binding domain, which also functions as the oligomerization domain, is usually located at the C-terminus of the protein. Upon binding of an effector molecule at the C-terminal domain, a conformational change occurs in the protein affecting the DNA-binding properties of the regulator. These conformational changes will result in repression or activation of gene transcription (Van Aalten et al., 2001). The C-terminal regulatory ligand binding domain differs significantly among individual proteins and is currently used to classify members of this superfamily into six major families: HutC, MocR, YtrA, AraR, PlmA, and FCD (FadR C-terminal Domain; Rigali et al., 2002; Lee et al., 2003; Franco et al., 2006; Zheng et al., 2009). Although the tertiary structures of several members of these large families are well described and publically available, the information on their sensory ligands and ligand binding pockets is very scarce. The FCD family clusters more than 40% of all GntR members, with FadR from *E. coli*, the regulator for fatty acid biosynthesis and degradation, being one of the best characterized (Van Aalten et al., 2000, 2001).

**Figure 1:**
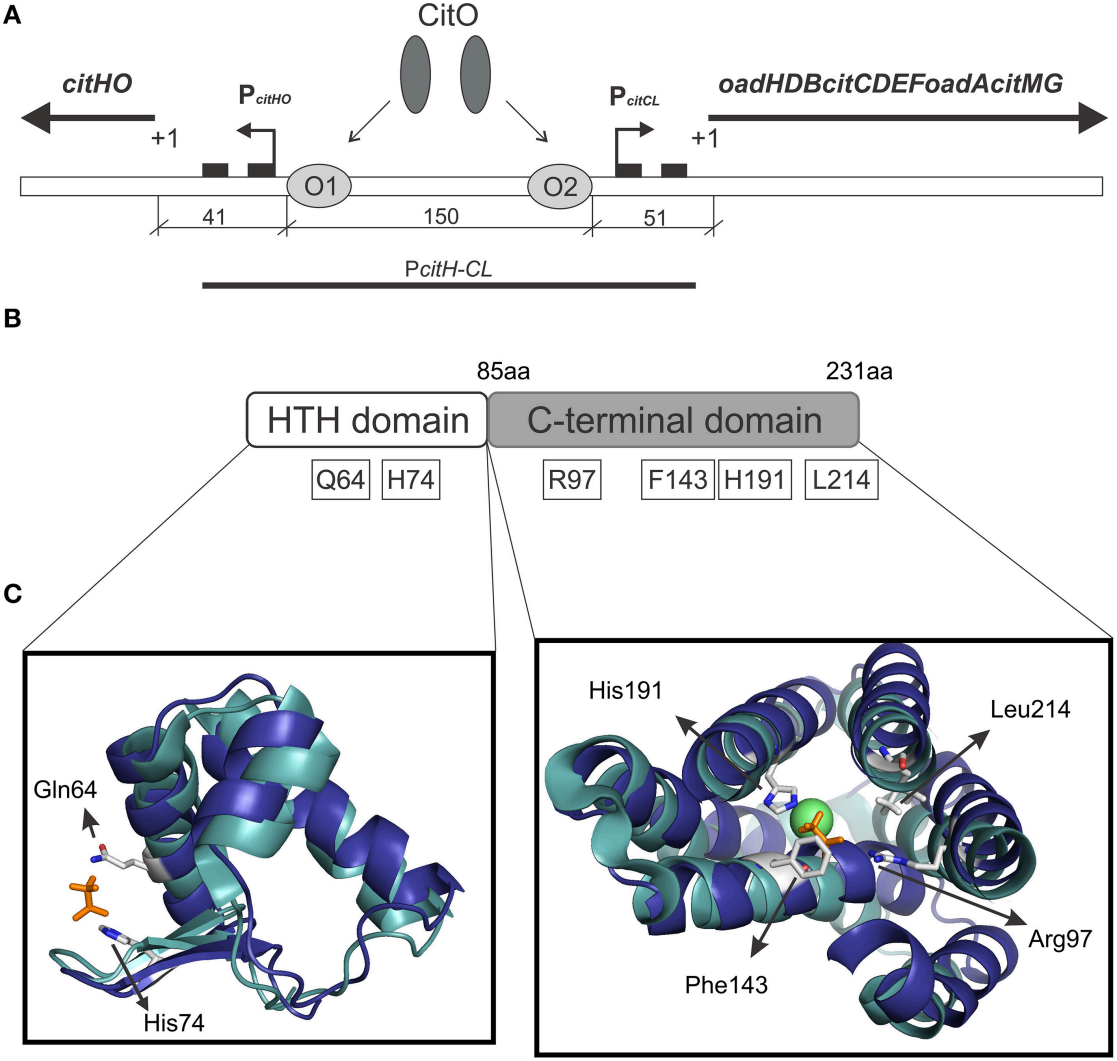
**Graphical representation of the citrate cluster and the domain organization in CitO and its predicted ligand binding pockets. (A)** Schematic representation of *cit* operons. *P*_*citHO*_ and *P*_*citCL*_ indicate promoter regions, +1 transcriptional start site, black rectangles indicate −10 and −35 boxes, O_1_ and O_2_ CitO binding sites. **(B)** The location of the amino acids selected in CitO for site-directed mutagenesis is indicated. **(C)** Close view of the residues in CitO predicted to mediate interactions with citrate and Ni^2+^. CitO *in silico* modeling (blue) was performed using the structure of TM0439 from *Thermotoga maritima* (PDB 3FMS, Zheng et al., 2009, cyan) as the template. In a stick representation (orange) are shown the acetate molecules, while as a green sphere is shown the Ni^2+^ found in TM0439 structure. The model was visualized and analyzed using PyMol.

CitO, a member of the FCD Family, is the transcription factor required for the expression of the genes involved in citrate metabolism in *Enterococcus faecalis*. The *cit* cluster is encoded in two divergent operons: *citHO* encoding for the citrate transporter *citH*, and the regulatory protein *citO*. The catabolic enzymes are encoded upstream, in the minus strand, in the *oadHDB-citCDEFX-oadA-citMG* operon (Blancato et al., 2006, 2008; Espariz et al., 2011; Repizo et al., 2013). There are two binding elements for CitO in the intergenic region, which regulate the expression of genes involved in the transport and degradation of citrate. In presence of citrate, CitO binds to the *cis*-acting sequences located in the *cit* intergenic region. In the *citHO* operon coding strand, CitO protects the region between positions −41 and −65 (binding site O_1_); whereas in the *oadHDB-citCDEFX-oadA-citMG* operon, CitO protects the region between positions −51 and −74 (binding site O_2_; Figure 1A). We previously showed that the interaction with citrate increased the binding of CitO to the DNA, and that the CitO/citrate complex had higher affinity for the O_1_ than the O_2_ binding site. Interestingly, *in vivo* and *in vitro* experiments suggest that CitO is specifically activated only by citrate and not by other metabolic intermediates, such as malate, succinate, or even by its isomer, isocitrate (Blancato et al., 2008). However, the molecular mechanisms of such specificity were not investigated.

In the present study, we report the analysis of the binding affinities of *E. faecalis* CitO for citrate. The binding pocket in CitO was analyzed by site-directed mutagenesis and the critical amino acids involved in the ligand binding were identified. Furthermore, we found that binding of a divalent cations, Ni^2+^, or Zn^2+^ in the C-terminus of the protein improved CitO-citrate interactions.

## Materials and methods

### Bacterial strains and growth conditions

The bacterial strains and plasmids used in this study are listed in Table 1. *E. coli* strains were grown at 37°C under aerobic conditions in Luria-Bertani medium (LB) (Difco), or on LB agar plates. *E. coli* DH5α (Invitrogen, Carlslab, CA) was used to propagate the plasmids for protein purification and site-directed mutagenesis. *E. coli* strain BL21(DE3)-Star (Novagen, Gibbstown, NJ) was used to express CitO. When required, the medium was supplemented with ampicillin (100 μg/ml) or kanamycin (50 μg/ml). All antibiotics and chemicals were purchased from Sigma-Aldrich (St. Louis, MO).

**Table 1:** **Bacterial strains, and plasmids used in this study**.

**Strain, or plasmid**	**Genotype, or description**	**Reference, or source**
***E. coli***		
DH5α	F^−^ϕ80d/*lacZΔM15* Δ(*lacZYA-argF*) U169 *recA1 endA1 hsdR17* (r${}_{\text{K}}^{-}$, m${}_{\text{K}}^{+}$) phoA *supE44* λ- *thi- 1 gyrA96 relA1*	Invitrogen
BL21-Star (DE3)	F^−^*ompT hsdSB*(r${}_{\text{B}}^{-}$ m${}_{\text{B}}^{-}$) *gal dcm rne131* (DE3); Str^r^	Novagen
***E. faecalis***		
JH2-2 (TX4000)	Plasmid-free wild-type strain; Fus^r^ Rif^r^	Jacob and Hobbs, 1974
JHB1	JH2-2 *citO*::pmCitO; Em^r^	Blancato et al., 2008
JHB14	JHB1 carrying pTCV- P*citCL*; Em^r^, Km^r^	Blancato et al., 2008
JHB14-02	JHB14 carrying pBM02; Em^r^, Cm^r^, Km^r^	This work
JHB14-wt	JHB14 carrying pCitO; Em^r^, Cm^r^, Km^r^	This work
JHB14-64	JHB14 carrying pCitOQ64A; Em^r^, Cm^r^, Km^r^	This work
JHB14-74	JHB14 carrying pCitOH74A; Em^r^, Cm^r^, Km^r^	This work
JHB14-97	JHB14 carrying pCitOR97A; Em^r^, Cm^r^, Km^r^	This work
JHB14-143	JHB14 carrying pCitOF143A; Em^r^, Cm^r^, Km^r^	This work
JHB14-191	JHB14 carrying pCitOH191A; Em^r^, Cm^r^, Km^r^	This work
JHB14-214	JHB14 carrying pCitOL214A; Em^r^, Cm^r^, Km^r^	This work
**PLASMIDS**		
pET-28a	Expression vector for protein purification; Km^r^	Novagen
pBM02	Shuttle vector for gene expression in LAB; Amp^r^, Cm^r^	Marelli and Magni, 2010
pTCV-lac	Promoterless vector which allows *lacZ* fusion construction; Km^r^	Poyart and Trieu-Cuot, 1997
pET-*citO*	pET28a derivative expressing His_6_-CitO	Blancato et al., 2008
pCitO	pBM02 derivative for expressing *citO* in *E. faecalis*	Blancato et al., 2008
pTCV-P*citCL*	pTCV-lac carrying *citCL* promoter	Blancato et al., 2008
pET-*citO*Q64A	pET-*citO* with CitO Q64 → A	This work
pET-*citO*H74A	pET-*citO* with CitO H74 → A	This work
pET-*citO*R97A	pET-*citO* with CitO R97 → A	This work
pET-*citO*F143A	pET-*citO* with CitO F143 → A	This work
pET-*citO*H191A	pET-*citO* with CitO H191 → A	This work
pET-*citO*L214A	pET-*citO* with CitO L214 → A	This work
pCitOQ64A	pCitO with CitO Q64 → A	This work
pCitOH74A	pCitO with CitO H74 → A	This work
pCitOR97A	pCitO with CitO R97 → A	This work
pCitOF143A	pCitO with CitO F143 → A	This work
pCitOH191A	pCitO with CitO H191 → A	This work
pCitOL214A	pCitO with CitO L214 → A	This work

*Amp^r^, Km^r^, Em^r^, Cm^r^, Fus^r^, Rif^r^, and Str^r^ indicate resistant to ampicillin, kanamycin, erythromycin, chloramphenicol, fusidic acid, rifampicin, and streptomycin, respectively*.

*E. faecalis* strains were routinely grown at 37°C without shaking in 100 ml sealed bottles filled with 20–50 ml of LB medium containing 0.5% w/v glucose. Erythromycin (5 μg/ml), kanamycin (1000 μg/ml), or chloramphenicol (10 μg/ml) were added when appropriate.

### DNA manipulations and gene cloning

Standard methods were used for chromosomal DNA isolation, restriction enzyme digestion, agarose gel electrophoresis, ligation, and transformation (Sambrook and Russell, 2001). Plasmids were isolated using spin miniprep kits (Qiagen, Valencia, CA) and PCR products were purified using QIAquick purification kits (Qiagen). All the primers used for this study are described in Table 2. The plasmids for protein purification, protein expression in *E. faecalis*, and transcriptional fusions are previously described in Blancato et al. (2008).

**Table 2:** **Oligonucleotides used in this study**.

**Primer**	**Sequence (5′ → 3′)**
**EMSA PROBE**	
CitOE-F	GTGTGAGAATATACAAACTTTCGCAG
CitOE-R	GGTATACGTTCATTATAGAAAAAACCG
**SITE DIRECTED MUTAGENESIS**	
CitOQ64A-F	GACGCCCATTCGCTTTGCTTTAGCAGAATTGGTCAAAGAACAATTG
CitOQ64A-R	CAATTGTTCTTTGACCAATTCTGCTAAAGCAAAGCGAATGGGCGTC
CitOH74A-F	GTCAAAGAACAATTGGTGGAAGCTATACCTATGGTGGGTATCGTG
CitOH74A-R	CACGATACCCACCATAGGTATAGCTTCCACCAATTGTTCTTTGAC
CitOR97A-F	GCTTATGAAATTTATGATATTGCTAAATCTTTGGACACTTTAGC
CitOR97A-R	GCTAAAGTGTCCAAAGATTTAGCAATATCATAAATTTCATAAGC
CitOF143A-F	GTAGATGACTTACTACAGAACGCTTCAGATTTTAATTCCTTTATTTATAC
CitOF143A-R	GTATAAATAAAGGAATTAAAATCTGAAGCGTTCTGTAGTAAGTCATCTAC
CitOH191A-F	CGTAGTATTGCCCTAGAAGAAGCTTGGTTAATTTTCCGCG
CitOH191A-R	CGCGGAAAATTAACCAAGCTTCTTCTAGGGCAATACTACG
CitOL214A-F	CACTTTTAACCCATGAACATGCAAATCGTTCGCTTCAATTTATTTTG
CitOL214A-R	CAAAATAAATTGAAGCGAACGATTTGCATGTTCATGGGTTAAAAGTG

Site-directed mutagenesis was performed using the QuikChange Site-directed Mutagenesis kit (Stratagene) according to the manufacturer's protocol. Plasmids pET-*citO* or pCitO were used as the templates. All the selected amino acids were changed to alanine. The mutations were verified by DNA sequencing using T7 universal primers.

### Protein purification

The His-tagged proteins in pET-*citO* were overexpressed in *E. coli* BL21-Star(DE3) (Novagen). The cells were grown in LB broth at 37°C to an OD_600_ = 0.6. Gene expression was induced with 0.4 mM isopropyl 1-thio-β-D-galactopyranoside (IPTG) and the cells were further incubated at 17°C for 16 h. The cells were then collected by centrifugation and stored at −80°C. For protein purification, the cells were resuspended in binding buffer (500 mM NaCl, 5% glycerol, 50 mM Tris pH 8.0, and 5 mM imidazole) with 1 mM phenylmethylsulfonyl fluoride and disrupted using a French pressure cell press. The lysates were clarified by centrifugation (30 min at 17,000 g) and applied to a metal chelate affinity column charged with Ni^2+^. The column was washed (in binding buffer with 15 mM imidazole) and the proteins were eluted from the column in elution buffer (binding buffer with 250 mM imidazole). The purified proteins were dialyzed against 10 mM Tris (pH 8.0), 500 mM NaCl, and 2.5% glycerol; aliquots were kept at −80°C.

### Electrophoretic mobility shift assays

EMSA analysis of CitO was performed using proteins purified according to the procedures described above. A fragment of 246 bp containing CitO binding sites (Blancato et al., 2008) was generated by PCR using biotin labeled (5′-end) primers (Table 2) and subsequently purified using QIAquick spin columns (Qiagen). The optimized mix for EMSA (20 μl) contained 1 ng of a 5′-labeled DNA fragment, 10 mM Tris-HCl (pH 7.5), 150 mM KCl, 0.5 mM EDTA, 0.2 mM DTT, 0.1% Triton X-100, 12.5% glycerol, 25 ng/μl Poly(dI-dC) nonspecific competitor DNA, and where indicated, purified CitO protein (0–400 nM) or citrate (0–5 mM). After incubation for 15 min at 37°C samples were separated on 5% acrylamide/bis-acrylamide non-denaturing gels, in 0.5X Tris borate-EDTA buffer, with a pH 7.5 (TBE). Electrophoresis was performed at 4°C. The DNA was then transferred from the polyacrylamide gel to an Amersham Hybond-N^+^ Membrane (Ge Healthcare, USA) by electroblotting at 250 mA for 45 min in a semidry transfer (Fisher Scientific, Pittsburgh, PA, USA). Transferred DNA was UV-crosslinked and biotin labeled DNA was detected using a Phototope-Star Detection kit (NEB). Membranes were exposed to Kodak X-ray film.

### Size exclusion chromatography

Size exclusion chromatography was performed using 200 μl protein samples. Aliquots contained 28.4 μM CitO, and where indicated, 1 mM citrate (pH 8.0). Following 30 min of incubation on ice, samples were injected onto a prepacked Superdex 75 (GE Healthcare, Sweden) gel filtration column connected to a LCC-501 plus (Pharmacia Biotech Inc., Piscataway, NJ) equilibrated with 50 mM Tris (pH 7.6), 300 mM NaCl, and 5% glycerol. Filtration was carried out at 4°C, using a flow rate of 0.5 ml/min. The eluted proteins were monitored continuously for absorbance at 280 nm using a UV-M II monitor (Pharmacia Biotech Inc.). Blue dextran 2000 was used to determine the void volume of the column. A mixture of protein molecular weight standards from Bio-Rad containing bovine serum albumin (66 kDa), albumin (45 kDa), lactalbumin (14.2 kDa), cytochrome C (12.7 kDa), and vitamin B12 (1.35 kDa) were also applied to the column under similar conditions. The elution volume and molecular mass of each protein standard was then used to generate a standard curve from which the molecular weight of eluted proteins was determined.

### Analysis of CitO associated metals

Metal-content was determined using inductively coupled plasma atomic emission spectrometry (ICP/AES) at the IFAS Analytical Research Laboratory, University of Florida, Gainesville.

### Differential scanning fluorimetry

The assay was carried out as described earlier (Pagliai et al., 2010). Briefly, CitO (10 μM), in presence or absence of increasing concentrations of citrate or malate (the latter was used as a negative control), was placed (in duplicate) into 96-well plates (Bio-Rad, Hercules, CA) and heated from 25 to 80°C at the rate of 1°C per min. The unfolding of the protein was monitored by the increase in fluorescence of the fluorophore SYPRO Orange (Invitrogen). Fluorescence intensities were plotted against temperature for each sample well and transition curves were fitted with the Boltzmann equation using Origin 8 software (Northampton, MA). The midpoint of each transition curve was calculated and values obtained in presence of the ligand compared to those calculated for CitO in absence of the ligand.

### Isothermal titration calorimetry

Measurements were performed on a VP-Microcalorimeter (MicroCal, Northampton, MA) at 25°C. The proteins were thoroughly dialyzed against 10 mM Tris (pH 8.0), 500 mM NaCl, and 2.5% glycerol. A solution of citrate (1 mM, pH 8.0) was directly prepared in dialysis buffer. Each titration involved a series of 3 μl injections of the effector molecule into the protein solution. The mean enthalpies measured from injection of the ligand in the buffer were subtracted from raw titration data prior to data analysis with Origin 8 software (MicroCal). Titration curves were fitted by a nonlinear least squares method to a function for the binding of a ligand to a macromolecule (Wiseman et al., 1989). From the curve thus fitted, the parameters Δ*H* (reaction enthalpy), *K*_*A*_ (binding constant, *K*_*A*_ = 1/*K*_*D*_), and *N* (reaction stoichiometry) were determined. From the values of *K*_*A*_ and Δ*H*, the changes in free energy (Δ*G*) and entropy (Δ*S*) were calculated with the following equation: Δ*G* = −RT ln*K*_*A*_ = Δ*H*−TΔ*S*; where R is the universal molar gas constant and T is the absolute temperature.

### Structure modeling and analysis

Phyre2 (http://www.sbg.bio.ic.ac.uk/phyre2/html/page.cgi?id=index; Kelley et al., 2015) was used to model the tridimensional structure of CitO. PDB entries 3FMS and 3C7J were retrieved as the highest scoring templates. PDB 3FMS corresponds to the protein TM0439, a putative transcriptional regulator from *Thermotoga maritima* (Zheng et al., 2009), while PDB 3C7J corresponds to a transcriptional regulator from *Pseudomonas syringae* pv. tomato str. DC3000. The structural models were visualized using the PyMOL molecular graphics system v1.3 (http://www.pymol.org/, Schrödinger, LLC).

### β-galactosidase assays

Overnight cultures of *E. faecalis* cells transformed with a reporter plasmid and/or a control plasmid grown in LB glucose containing containing kanamycin, erythromycin, and chloramphenicol were diluted to an OD_600_ = 0.08 and subsequently cultured in fresh LB medium supplemented with or without 17 mM citrate. At early stationary phase (OD_600_ = 0.8), the cells were permeabilized with 0.15% sodium dodecyl sulfate (SDS) and 1.5% chloroform in Z-buffer (60 mM Na_2_HPO_4_, 40 mM NaH_2_PO_4_, 10 mM KCl, 1 mM MgSO_4_, 50 mM β-mercapthoethanol). β-galactosidase activity was colorimetrically determined using ONPG as a substrate at 420 nm. The activity was quantified as described by Miller (1972), and subsequently normalized to the CitO protein concentration determined by western blot, and expressed as arbitrary units (AU). The assays were performed in triplicate. The reporter plasmid, pTCV-P*citCL* (Table 1) (Blancato et al., 2008), was used to determine P_*citCL*_ promoter activity. The basal AU was determined from assays performed with the empty plasmids (pBM02 and pTCV-*lac*, Table 1).

### Western blot analysis

*E. faecalis* strains were grown at 37°C for 6 h in LB medium in the presence or absence of 17 mM citrate. Cells were harvested by centrifugation and the cell free extracts were prepared by vortexing cells with glass beads (425–600 microns, Sigma-Aldrich). Proteins were separated by sodium dodecyl sulfate-polyacrylamide gel electrophoresis (SDS-PAGE) on a 12% polyacrylamide gel, and transferred to a nitrocellulose membrane by electroblotting. Proteins were detected using primary antibody rabbit polyclonal antisera against CitO of *E. faecalis*. Antibodies were visualized by using a secondary anti-rabbit antibody coupled to alkaline phosphatase (Bio-Rad).

## Results

### Functional analysis of the CitO ligand binding pocket

We previously established that CitO induced the expression of the *cit* operons by specifically binding citrate (Blancato et al., 2008). To determine the citrate binding site, a structural model of *E. faecalis* CitO was constructed using Phyre2. The structure of TM0439 from *T. maritima* (PDB 3FMS, Zheng et al., 2009) was retrieved as the highest scoring template (Figure 1). The obtained model had a 90% of coverage, a QMEAN Z-score of −2.97 (Benkert et al., 2011), and 19% identity with TM0439 (Supplementary Figure S1). The structure of TM0439 was solved with two molecules of acetate; one localized in the N-terminus and the second in the C-terminus next to a Ni^2+^ atom. We performed a structural alignment between CitO and TM0439, with the associated acetate molecules used as an indication of the potential binding pocket for citrate in CitO. Although the C-terminus is associated with the binding of effector molecules, the binding site located in the HTH domain was not discarded in our studies. To determine which pocket is the biologically active site, the relevant residues surrounding the acetate molecules were selected for further analyses (Figures 1B,C). In the HTH domain, amino acids Q64 and H74 were selected. In the C-terminal domain, six residues within 3 Å to the acetate molecule were identified (R97, F143, N147, H191, H213, and L214). Specifically, residues N147, H191, and H213 were located in the proximity of the acetate molecule and in close distance to a Ni^2+^ atom associated with the protein. Based on these observations, we hypothesized that a cation may facilitate the binding of the ligand to the protein or be involved in shaping the binding groove. The residues Q64, H74, R97, F143, H191, and L214 were mutated to alanine and the proteins were subsequently purified. The effect of each individual mutation was then evaluated based on their ability to bind DNA and/or citrate, and their biological relevance was validated *in vivo*.

**Figure 2:**
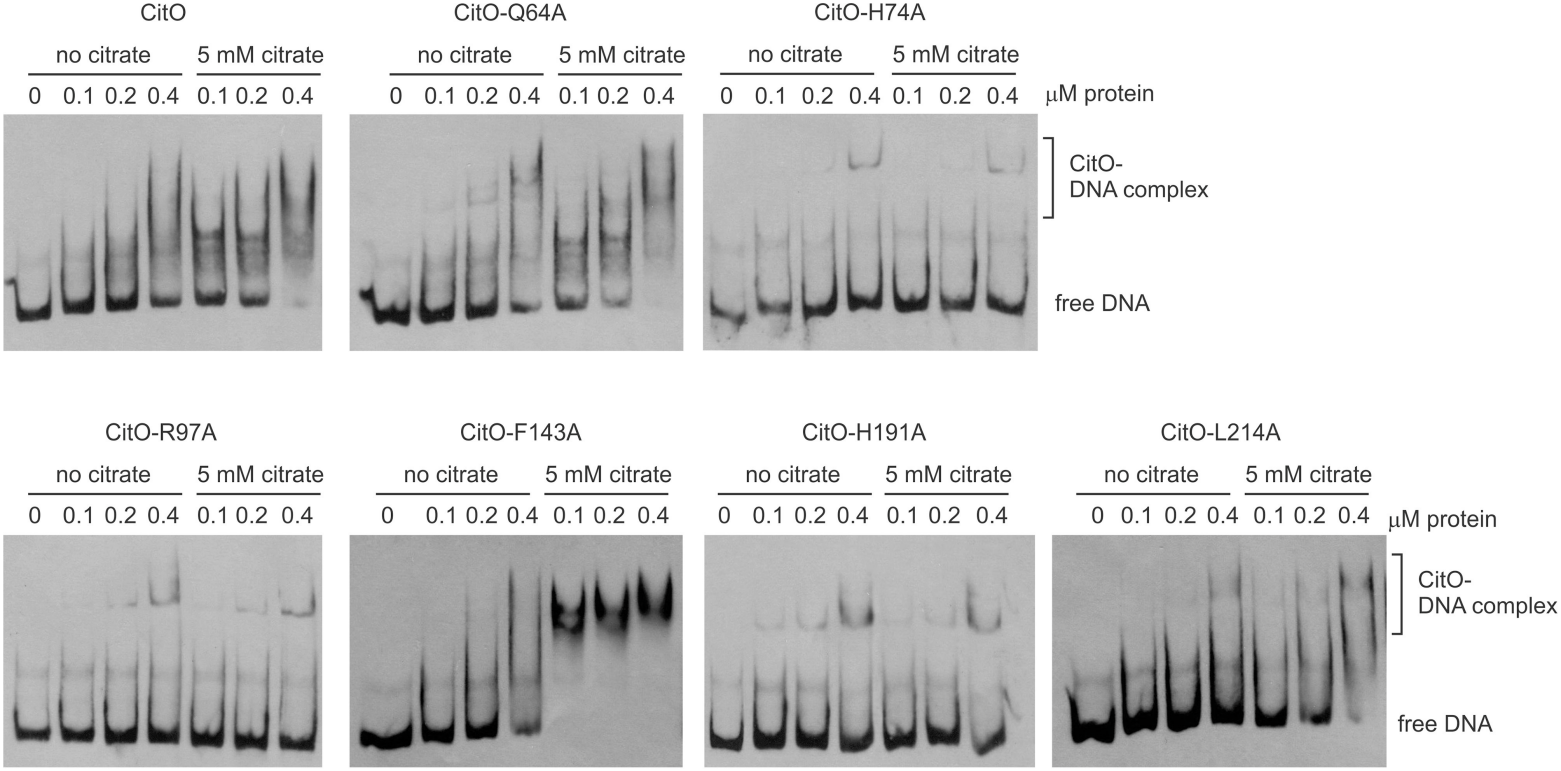
**Alanine substitution on the HTH or C-terminal domains of CitO decrease binding to *P*_*citH*−*CL*_**. EMSA were performed using biotin-labeled *P*_*citH*−*CL*_ (1 ng) and increasing concentrations of CitO-wt or CitO mutants (0.1, 0.2, 0.4 μM), in the presence or absence of citrate (5 mM) as indicated on top of each panel. No protein was added to the first lane.

The ability of CitO or its mutant variants to bind DNA was determined using a 246 bp fragment (*P*_*citH*−*CL*_) containing the promoter region of the *citHO* operon (*P*_*citH*_) and the *citCL* operon (*P*_*citCL*_) harboring both the O_1_ and O_2_ binding sites (Figure 1A). It was observed that the binding of CitO wild type protein (CitO-wt) to this region resulted in multiple unstable complexes. The stability of the CitO-*DNA* complexes increased with CitO concentrations (up to 400 nM) and with the addition of citrate (up to 5 mM) to the binding reaction (Figure 2). Although several binding buffers were tested, a defined band for the protein-DNA complex was not obtained.

Once the binding conditions for CitO-wt were optimized, the ability of mutants in CitO to bind DNA was evaluated. The results obtained with EMSA (Figure 2) allowed the classification of mutations in four groups: group I showed no effect on DNA or citrate binding (Q64A). Group II showed both a decrease in DNA binding and less response to citrate (H74A, R97A, and H191A). Group III showed a decrease in DNA binding but was able to increase DNA binding in the presence of citrate (L214A). Group IV showed DNA binding comparable to CitO-wt in absence of citrate, but it had a dramatic increase in DNA binding when citrate was added to the reaction mix (F143A).

The mutation Q64A (located in the DNA binding domain) showed a similar DNA and ligand binding pattern compared to CitO-wt. CitO-Q64A showed some DNA binding at 200 nM protein. In presence of citrate, the ability of this protein to bind the DNA probe used was comparable to the wild type. These results suggest that Q64 is not critically involved in DNA or citrate binding.

The mutations on H74A (located in the DNA binding domain), R97A, and H191A (located in the C-terminal domain) severely affected the DNA binding. With CitO-H74A, a faint protein:DNA complex was observed only after 200 nM protein was used. Meanwhile, with the proteins carrying the mutations R97A or H191A, the complex was detectable at the lowest concentration of protein used (100 nM). The addition of citrate did not modify the formation of protein:DNA complexes when compared with CitO-wt (Figure 2). These results indicate that H74, R97, and H191 amino acids could be involved in the interaction with DNA, citrate, or both.

The L214A mutation (located in the C-terminal domain) showed decreased DNA binding compared to CitO-wt. The addition of citrate increased the binding of CitO-L214A to *P*_*citH*−*CL*_ to a level similar to CitO-wt (Figure 2).

CitO-F143A, a mutation located in the C-terminal domain, showed a similar DNA binding pattern to CitO-wt in absence of citrate. Surprisingly, in presence of 5 mM citrate a strong shift in the CitO-F143A was observed, suggesting higher affinity of the protein for the ligand (Figure 2).

### Mutations in the C-terminal of CitO modify its thermal stability in the presence and in the absence of citrate

Gel shift assays indicated that specific amino acid substitutions affected the ability of CitO to bind DNA, as well as the binding of the effector molecule citrate. To identify the residues involved only in citrate binding, differential scanning fluorimetry (DSF) followed by isothermal titration calorimetry (ITC) analyses were performed.

**Figure 3:**
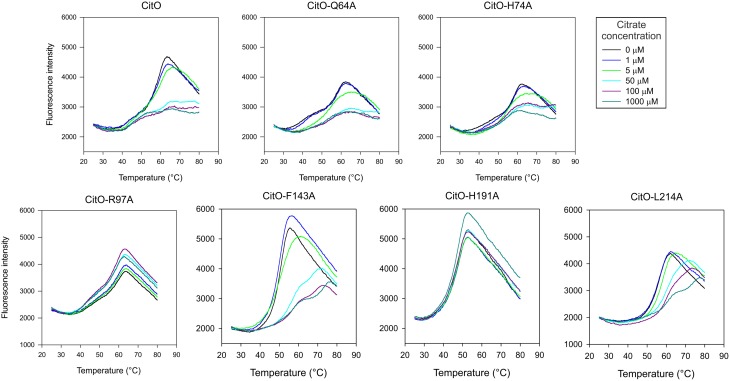
**Mutations in the C-terminal of CitO modify its thermal stability in the presence and in the absence of citrate**. DSF was performed with purified CitO-wt and its mutant variants (indicated on top of each figure) in the absence or presence of increasing concentrations of citrate (1, 5, 50, 100, or 1000 μM). Purified proteins (20 μM) were subjected to gradually increasing temperatures in the presence of SYPRO Orange fluorophore. Fluorescence intensities were plotted against temperature and transition curves were fitted using the Boltzmann equation.

DSF was carried out by monitoring the increment in fluorescence of SYPRO orange due to protein unfolding. The proteins were subjected to DSF in presence and absence of ligands. CitO-wt showed two apparent melting temperatures (*Tm*) in DSF analyses (Figure 3): *Tm*_1_ = 43°C and *Tm*_2_ = 58°C. This effect may result from the sequential unfolding of the different protein domains or changes in oligomerization of the protein (Niesen et al., 2007; Gupta and Grove, 2014). The predicted molecular weight of the CitO monomer is 27 kDa. Size-exclusion chromatography analysis indicated that CitO has an apparent molecular weight of 48 kDa in solution, consistent with the predicted molecular weight of the dimer (Supplementary Figure S2). The addition of citrate did not modify the oligomerization state of CitO in solution. Altogether, these data indicate that different transition temperatures observed for CitO-wt may be the result of the subsequent and independent unfolding of DNA and ligand-binding domains. In agreement with this observation, the addition of citrate to CitO-wt induced a shift in the *Tm*_2_ but not on *Tm*_1_, indicative of increased protein stability upon ligand binding (Figure 3). The specificity of this interaction was determined by adding other small carboxylic acids, such us malate, succinate, or isocitrate. As expected, CitO did not show a shift in the *Tm*_2_ in presence of other small molecules. Since changes in *Tm*_2_ were associated with citrate binding to CitO, this parameter was analyzed in all the CitO mutants.

CitO-Q64A melting profile was similar to CitO-wt, the addition of 5 μM citrate resulted in a clear shift in *Tm*, while at 50 μM a higher stability was observed. Interestingly, the CitO-H74A mutant showed a melting profile similar to CitO-Q64A and it was comparable to the wild type. These results indicate that CitO-H74A is still able to interact with citrate in solution. Since CitO-H74A binds DNA with low affinity, we hypothesized that this residue is involved in direct binding to DNA and/or in signal transduction from the C-terminal domain to N-terminal domain.

CitO-R97A and CitO-H191A also showed poor DNA and citrate binding on EMSA (Figure 2). DSF assays showed that in the presence or absence of citrate the protein denatured at similar temperature, even at high citrate concentrations (1 mM). These results suggest that CitO-R97 and CitO-H191 are most likely, directly involved in citrate binding.

The CitO-L214A mutant showed an increase in *Tm*_2_ (Δ*Tm* = 4°C) only when 50 μM citrate (or higher) was present in the mixture. The CitO-F143A mutant also showed one transition curve with a *Tm* = 50°C. In agreement with the results obtained in EMSA, the addition of 50 μM citrate induced a strong shift in the *Tm* (*Tm* = 57°C). These results suggest stronger binding of the effector molecule in the binding pocket of the regulator.

**Figure 4:**
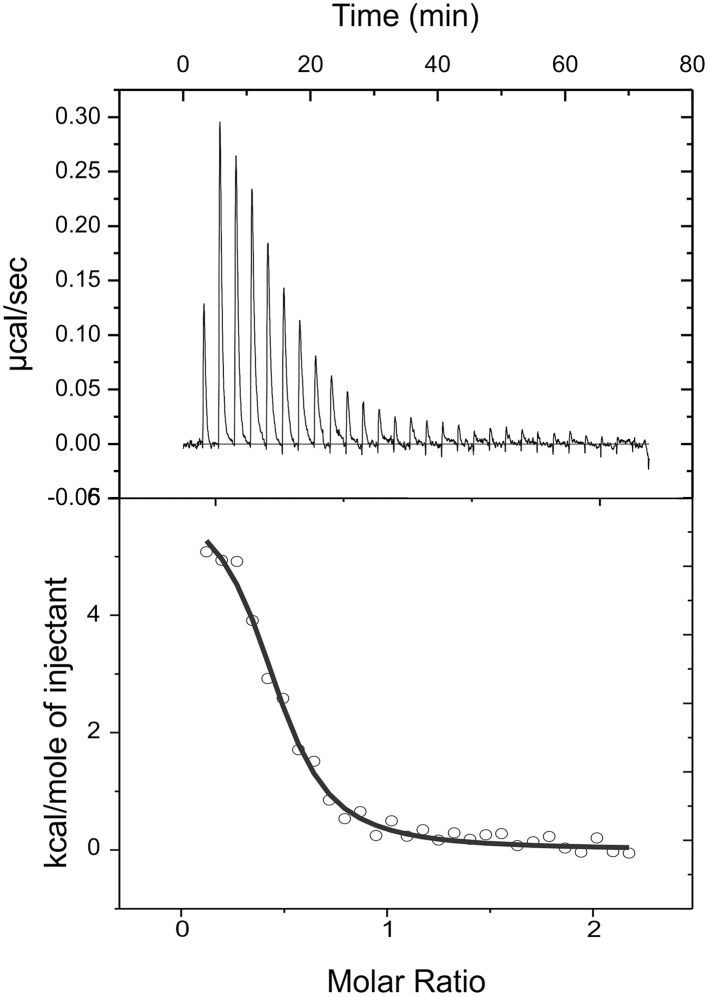
**Isothermal titration calorimetric data for the binding of citrate to CitO**. Heat changes (upper panel) and integrated peak areas (lower panel) for the injection of a series of aliquots (3 μl) of 1 mM ligand in a solution of 28 μM CitO. Experiments were carried out at 25°C.

To further characterize the interactions of CitO and its mutated variants with the effector molecule, the thermodynamic properties of CitO interactions with citrate were determined using ITC. The titration of CitO with citrate followed an endothermal heat change profile, giving rise to a sigmoidal binding curve (Figure 4). The data were fitted with the Origin software using the “one set of sites model.” The estimated thermodynamic parameters are summarized in Table 3. CitO-wt dissociation constant (*K*_*D*_) with citrate was in the low micromolar range (*K*_*D*_ = 1.2 ± 0.2 μM) indicating a high affinity for the ligand. Interestingly, the affinity for citrate increased in the CitO-F143A mutant as evidenced by lower *K*_*D*_ values (Table 3). The Δ*H* was positive indicating an entropically driven reaction and the enthalpy variations were similar in the wt and all of the mutants (~6000 cal/mol) with the exception of CitO-F143A, which showed a Δ*H* = 16020 cal/mol. In agreement with DSF assays, CitO-R97A and CitO-H191A mutants did not interact with citrate.

**Table 3:** **Thermodynamic parameters derived from the calorimetric titration of CitO variants with citrate**.

**Protein**	***K_*D*_*(μM)**	***N***	**Δ*H* (cal/mol)**	**TΔ*S* (cal/mol/deg)**	**Δ*G* (cal/mol)**
CitO-wt	1.2 ± 0.2	0.44	6122	47.3	−7983.4
CitO-Q64A	0.77 ± 0.1	0.27	6724	50.5	−8340
CitO-H74A	0.82 ± 0.04	0.33	6500	50.2	−8434.6
CitO-R97A	ni^*^	ni^*^	ni^*^	ni^*^	ni^*^
CitO-F143A	0.44 ± 0.08	0.34	16020	82.9	−8685.7
CitO-H191A	ni^*^	ni^*^	ni^*^	ni^*^	ni^*^
CitO-L214A	1.2 ± 0.2	0.123	5780	46.5	−8070

* *no interaction*.

The stoichiometry of the reaction was *N* = 0.4. Considering that CitO is a dimer in solution, these results suggest either the binding of one citrate molecule per dimer or that a fraction of the purified protein may contain citrate bound picked up during the heterologous expression in *E. coli* [citrate concentration in *E. coli* ranges 2–30 mM depending on growth conditions (Bennett et al., 2009)].

### Citrate- CitO interactions are mediated by a metal cofactor

TM0439 has three histidines (His134, His174, and His196) in its C-terminal domain, with imidazole groups arranged in a three-blade propeller scaffold. This arrangement is usually associated with the binding of metal groups like Zn^2+^ or Ni^2+^ with high affinity. In agreement with this observation, TM0439 is able to bind these ions with *K*_*D*_ values in the nanomolar range (Zheng et al., 2009). Based on the structural model, the CitO residues potentially involved in metal (Me^2+^) binding were Asn147, His191, and His213. IPC-AES analysis was performed to determine if a metal atom was found in CitO-wt, or some mutant variants with different responses to citrate. It was found that CitO-wt, CitO-R97A, and CitO-F143A contained 1.02, 1.1, and 1.06 atoms of Ni^2+^ per molecule of protein, respectively. In contrast, in the mutant CitO-H191A, the amount of metal decreased to 0.59 Ni^2+^ per molecule of protein. These results indicate that H191 is likely involved in metal interaction. Trace amounts of Zn^2+^ (0.05–0.07 Zn^2+^ per molecule of protein) were also found in all the tested proteins.

**Figure 5:**
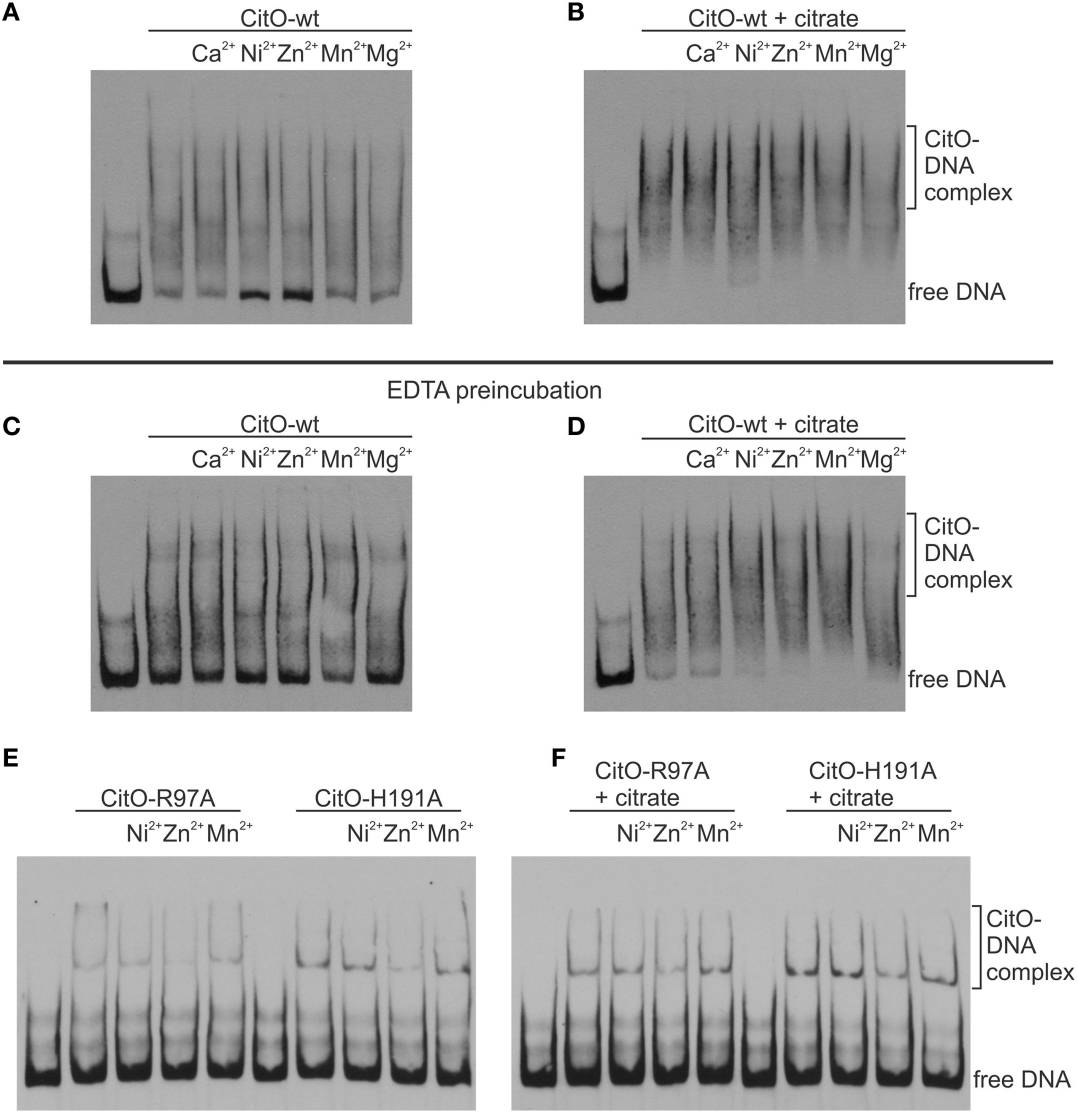
**Metal binding to CitO promotes binding to *P*_*citH*−*CL*_ in presence of citrate**. EMSA were performed using biotin-labeled DNA (1 ng); 0.2 μM CitO-wt **(A–D)**, CitO-R97A, or CitO-H191A **(E,F)**; Me^2+^ (2 mM) in the absence **(A,C,E)** or presence of 5 mM citrate **(B,D,F)**. CitO used for **(A)** and **(B)**, was incubated in buffer and then dialyzed, whereas the CitO-wt or mutants shown in **(C–F)** were incubated with EDTA for 2 h at 4°C and then dialyzed over night before conducting EMSAs.

The change of H191 to Ala caused a strong effect on the ability of CitO to bind DNA and interact with citrate. These results may be explained by the reduced ability of the CitO-H191A mutant to bind Me^2+^. To test this hypothesis, the DNA binding ability of CitO to *P*_*citH*−*CL*_, supplemented with different divalent cations, was evaluated. It was found that the addition of Ca^2+^, Mn^2+^, or Mg^2+^ did not modify the binding of CitO to *P*_*citH*−*CL*_, while Zn^2+^ and Ni^2+^ decreased the binding (Figure 5A). In the presence of citrate, none of the Me^2+^ affected the binding of CitO to *P*_*citH*−*CL*_ (Figure 5B).

To test the role of Me^2+^ in citrate binding, CitO was incubated in presence of 100 mM EDTA. It was found that the depletion of Me^2+^ decreased the binding of CitO to *P*_*citH*−*CL*_; however, just the addition of metals did not rescue the phenotype observed (Figure 5C). Moreover, it was found that the addition of citrate alone improved the formation of intermediate complexes, while the supplementation with citrate and Ni^2+^ or Zn^2+^ increased the stability of the larger CitO-*P*_*citH*−*CL*_ complexes (Figure 5D). These results indicate that citrate binding to CitO is mediated by a metal cofactor, and that these interactions are required for CitO binding to *P*_*citH*−*CL*_.

These results were confirmed by testing the mutants CitO-R97A, which is irresponsive to citrate but it is still able to bind metal; and CitO-H191A, which is irresponsive to citrate and it has decreased amounts of bound metal. As expected, the addition of citrate or metals did not increase the binding of both mutant proteins to *P*_*citH*−*CL*_ (data not shown). The pretreatment of CitO-R97A and CitO-H191A with EDTA showed similar results (Figures 5E,F).

### Mutations in the CitO citrate binding pocket reduced *in vivo* expression of *P*_*citCL*_

**Figure 6:**
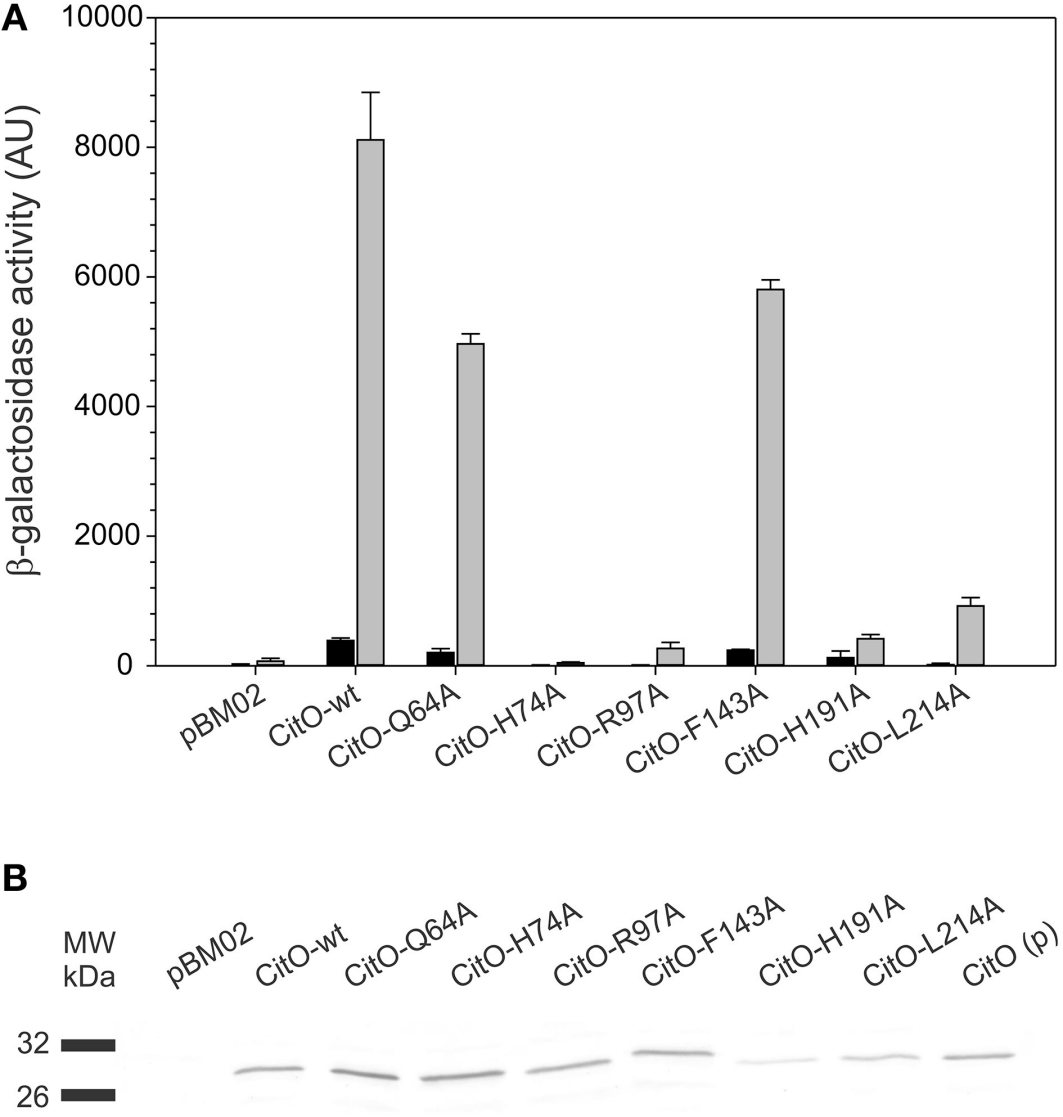
**Amino acid substitutions in CitO affect the modulatory activity of CitO on promoter activity. (A)** pBM02 derived plasmids were used to express constitutively CitO and its variants in a *citO* deficient strain harboring pTCV-P*citCL* reporter plasmid. Cells were grown in the presence (gray bars) or absence (black bars) of 17 mM citrate, and assayed for β-galactosidase activity (expressed in arbitrary units, AU) after 6 h of growth. **(B)** The quantification of the protein levels by Western blot, using polyclonal antibodies against CitO, was performed by densitometry in order to calculate specific β-galactosidase activity. CitO(p) 0.1 μg of purified CitO used as a positive control.

The effect of mutations in CitO was determined *in vivo* by using a *P*_*citCL*_ fusion to *lacZ* (pTCV-P*citCL* plasmid, Table 1). *E. faecalis* JHB1 was transformed with pTCV-P*citCL* (Strain JHB14). Strain JHB14 was subsequently transformed with pCitO derived plasmids (Table 1), which allowed the constitutive expression of the wild type or mutant forms of CitO. Strain JHB14-02 carrying the empty pBM02 plasmid was used as a negative control (Table 1). The densitometry analysis of CitO immunoreactive bands (Figure 6B) was used to normalize the reporter activity.

It was found that in the absence of citrate, strains carrying CitO-Q64A, CitO-F143A had a two-fold reduction in the β-galactosidase activity compared to CitO-wt. Strain JHB14-191, carrying CitO-H191A, showed a four-fold reduction in the base level activity, while strains JHB14-74, JHB14-97, and JHB14-214 (carrying CitO-H74A, CitO-R97A, and CitO-L214A, respectively) showed activity levels similar to the empty plasmid control. These results are in agreement with the reduced DNA binding activity found in EMSA.

When LB was supplemented with citrate, the β-galactosidase activity was induced in the strains containing CitO-wt, CitO-Q64A, or CitO-F143A (Figure 6). These results are similar to the *in vitro* results obtained by EMSA and ITC. In contrast, strains carrying CitO-H74A, CitO-R97A, CitO-H191, or CitO-L214A showed a reduced β-galactosidase activity in presence of citrate.

The results obtained in CitO-H74A mutant suggest that modifications in the HTH domain introduced in CitO-H74A resulted in a reduced DNA and citrate binding *in vivo*. These results contrast the DNA binding with the ITC data, where citrate binds with similar affinities to CitO-H74A and CitO-wt. The apparent discrepancy can be explained by the potential involvement of H74 in signal transduction from the DNA to the ligand binding domain in CitO, and that its mutation affected the ability of CitO to transduce the signal between these domains.

Mutations in CitO on residues R97 and H191 showed very low β-galactosidase activity in presence of citrate. These results can be explained by the reduced DNA binding capabilities of these mutants and the reduced binding affinity of CitO-R97A and CitO-H191A for citrate observed *in vitro*. Based on the combined *in vitro* and *in vivo* results, we concluded that residues R97 and H191 in CitO mediate the binding to citrate.

In contrast to the low basal level activity observed in the CitO-L214A strain in absence of citrate, the addition of citrate to the growth media resulted in a large increment in the β-galactosidase activity (64-fold). These results are similar to those obtained in EMSA (reduced DNA binding to *P*_*citH*−*CL*_) and ITC (similar affinity for citrate as CitO-wt).

## Discussion

CitO is a transcriptional activator of the citrate metabolism in *E. faecalis*. It has been classified as a member of the GntR superfamily and clustered in the FCD family. The main characteristic of this subgroup of proteins was established based on the structural features of *T. maritima* TM0439. In this transcription factor, one nickel ion and two acetate molecules were present in the crystal structure of TM0439. Based on these findings, Zheng et al. (2009) suggested that the majority of proteins with FCD domains should be metal binding proteins. Contrary to traditional metal sensing transcription factors, such as ArsR, MerR, Fur, DtxR, or CodY, in TM0439 the metal is buried at the bottom of the ligand binding cavity. The buried location of the metal suggested an important contribution of the metal center in stabilizing the overall active pocket structure. However, the role of individual amino acids in the metal or carboxylic acid binding was not assessed. In order to gain further insight on the ligand-protein interactions within the FCD domains, CitO structure was modeled in the automated mode. The structure of *T. maritima* TM0439 (PDB 3FMS) and PDB 3C7J corresponding to a transcriptional regulator from *P. syringae* pv. tomato str. DC3000 were retrieved as the highest scoring templates. In TM0439, the location of two acetate molecules, one in the N-terminus and another in the C-terminus, generated uncertainties on which was the biologically relevant pocket. Although the biological role of the acetate molecules has not been determined for TM0439, their location proved instrumental in the identification of the citrate binding pocket in CitO.

Previous molecular data obtained with FadR from *E. coli* showed that acyl-CoA binds in the C-terminal domain (Raman and Dirusso, 1995; Van Aalten et al., 2000, 2001) showed that upon binding to the acyl-CoA molecule, the protein backbone undergoes dramatic conformational shifts throughout the main protein scaffold. This movement, typical of allosteric proteins, generates a rearrangement of the DNA binding domains. As a consequence of such movement, the DNA recognition helices become largely separated, preventing the interaction with the DNA molecule and consequently derepressing transcription. In order to discriminate which of the two putative citrate binding sites were active in CitO we performed site-directed mutagenesis at both sites. Mutations of CitO residues Q64 and H74 (located in the N-terminal domain) confirmed that CitO complies with the GntR paradigm, in which the N-terminal domain is involved in DNA binding and not in ligand interaction. Interesting, mutagenesis of residues in the C-terminal part of CitO affected both DNA and ligand binding.

In the C-terminal FCD domain, the CitO F143 residue was located in close proximity to the acetate molecule found in TM0439. The change of F143 to alanine did not affect DNA binding in the absence of citrate. Surprisingly, a dramatic increase in DNA binding was observed upon addition of citrate. This *in vitro* data suggested that F143 could act as a gate keeper thereby restricting or modulating access of citrate to its binding pocket. However, CitO-F143A was less effective to induce P_*citCL*_ activity *in vivo*. Another interesting result was obtained with a mutant in residue L214. Although the ligand binding was not affected in the CitO-L214A mutant *in vitro*, the mutation appears to be critical for protein function as evidenced by the very low transcriptional activation of the β-galactosidase reporter gene *in vivo*. Collectively, the results indicate that although some of the residues that form the ligand binding cavity are not involved in direct contact with citrate, they may have an important role in allowing the protein to acquire an optimal conformation for DNA binding.

Conversely, CitO residues R97 and H191 were found to be essential for both DNA and ligand binding as determined by ITC, EMSA, and in *in vivo* tests, suggesting that those residues are in direct contact with the citrate molecule. These two residues may also be involved in further signal transduction to the N-terminus, allowing the correct positioning of the HTH domain on the DNA. In the *E. coli* biofilm repressor McbR (PDB: 4PF9), although the ligand was not defined in the structure, the residues R89 (R97 in CitO), D135 (F143 in CitO), R139 (D147 in CitO), and D211 (Supplementary Figure S1) were indicated as involved in ligand binding (Lord et al., 2014). The structural model constructed for CitO indicated that H191 is part of an Asn147-His191-His213 triad involved in the coordination of a divalent cation. Accordingly, stoichiometric amounts of Ni^2+^ were found in CitO-wt samples, whereas the CitO-H191A mutant showed a strong reduction in Ni^2+^ concentrations. The decreased amount of metals in CitO-H191A correlated with a reduction on DNA and ligand binding affinities (Figures 2, 3, Table 3). These results suggest that the metal is important for protein function and that the remaining residues of the pocket (Asn147 and His213), perhaps with the aid of second sphere of coordination, could still bind Ni^2+^ but in a less efficient configuration. Furthermore, when the metal was removed from the CitO-wt, the protein was unable to sense the presence of the citrate (Figure 5D), indicating that both citrate and the metal are required for proper protein function.

The presence of metals in the GntR superfamily of transcriptional factors was initially reported in the repressor LldR from *Corynebacterium glutamicum* (PDB 2DI3) involved in L-lactate and fructose/glucose utilization. A Zn^2+^ ion was located in the structure at the core of the helical bundle with three coordinating histidine residues (His148, His196, and His218); however, the biological role of the cation in this protein was not determined (Gao et al., 2008). The metal binding site is conserved across other proteins containing the FCD domain, such as TM0349 (Zheng et al., 2009) and PS5454 from *P. syringae* (PDB: 3C7J; Supplementary Figure S1). These residues are also highly conserved in other sequence homologs of CitO found in *Streptococcus mutants, Streptococcus pyogenes, Lactobacillus casei, Lactobacillus sakei*, and *E. faecium* that are associated with the citrate fermentation pathways.

The binding of citrate in complex with a metal seems widespread in other families of transcription factors. Recently a citrate-Mg^2+^ binding pocket was reported for AcnR, a member of TetR family of transcriptional regulators. AcnR controls the expression of aconitase in *C. glutamicum* (Garcia-Nafria et al., 2013). Contrary to other TetR ligands, the citrate-Mg^2+^complex was found in a second binding site distant from the DNA-binding domain, with a *K*_*D*_ of 6 mM. The ligand was located with its apolar carbon atoms pointing to the hydrophobic interior and the polar atoms facing the outer side of the pocket. In AcnR, the magnesium ion is coordinated by three oxygens in the citrate, two water molecules, and the carboxyl oxygens of a glutamic acid residue (Garcia-Nafria et al., 2013).

In conclusion, previous structure-based evidence suggested that members of FCD family may act as metal-sensors or that the metal present in these domains would play a rather structural role while being required for interaction with other effector molecules. Based on the evidence presented here, we proposed that citrate interacts with the buried Ni^2+^ or Zn^2+^ molecule in the C-terminal domain, triggering conformational changes in CitO that reorient the N-terminal DNA binding domain, increasing the recognition of its specific DNA target.

## Author contributions

VB, CM, and GL conceived and designed the experiments. VB and FP performed the experiments. VB, CM, CG, and GL analyzed the data and wrote the paper.

### Conflict of interest statement

The authors declare that the research was conducted in the absence of any commercial or financial relationships that could be construed as a potential conflict of interest.

## Abbreviations

EMSA: Electrophoretic mobility shift assays
DSF: Differential scanning fluorimetry
ITC: Isothermal Titration Calorimetry
*Tm*: melting temperature
IPC-AES: inductively coupled plasma atomic emission spectrometry.
