# Supplementary material for: Functional Analysis of the Citrate Activator CitO from Enterococcus faecalis Implicates a Divalent Metal in Ligand Binding
Source: Front Microbiol. 2016 Feb 9;7:101. doi: 10.3389/fmicb.2016.00101 (PMC4746285; doi:10.3389/fmicb.2016.00101)
Supplement: Supplementary file 1 [file Image1.PDF]

Supplementary Material

Functional analysis of the citrate activator CitO from *Enterococcus faecalis* implicates a divalent metal in ligand binding

Víctor S. Blancato[1,2], Fernando A. Pagliai[2], Christian Magni[1], Claudio F. Gonzalez[2] and Graciela L. Lorca[2]*

[1]Laboratorio de Fisiología y Genética de Bacterias Lácticas, Instituto de Biología Molecular de Rosario, Consejo Nacional de Investigaciones Científicas y Técnicas (IBR-CONICET), Rosario, Santa Fe Argentina. [2]Department of Microbiology and Cell Science, Genetics Institute, Institute of Food and Agricultural Science, University of Florida, Gainesville, FL, USA.

Running Head: Identification of CitO citrate binding pocket

[*]Correspondence:
Dr. Graciela L. Lorca,
Department of Microbiology and Cell Science, Genetics Institute and Institute of Food and Agricultural Sciences,
University of Florida,
2033 Mowry Road, Room 307,
Genetics Institute 325E, P.O. Box 103610,
Gainesville, FL 32610- 3610, USA
Email: glorca@ufl.edu, Phone: (352) 273-8090

```
      1                                    α1                    43
CitO  ...MNPTVEA VKRNLDLTSS KP....LKIC TYEAFKKTII LGDIPAGERI
3FMS  .......... ........MK KIEVDLVRTK VYNLLKEMIL NHELKLGEKL
4P96  .......... .........M VIKAKSPAGF AEKYIIESIW NGRFPPGSIL
2HS5  MTSSNALRGD AHSRLAAHRG LLERTSRTTR VAGILRDAII DGTFRPGARL
3C7J  ...MRKSDRE AFLGSVLGNE QPPAHLARTV IEEKLRNAII DGSLPSGTAL
4P9F  .......... .....MPGTG KMKHVSLTLQ VENDLKHQLS IGALKPGARL
3IHU  .......... .MPIDTPTDA SPADGSASDT VFFGIMSGLE LGTFVPGQRL
1E2X  .......... .........M VIKAQSPAGF AEEYIIESIW NNRFPPGTIL
2DI3  .......... .......... ..MSVKAHES VMDWVTEELR SGRLKIGDHL

                                     HTH domain
                                      β1       β2
    44     α2          α3                                  α4   92
CitO  N.EKEFSEQL NISRTPIRFA LQELVKEQLV EHIPMVGIVV KGISMKDAYE
3FMS  N.VRELSEKL GISFTPVRDA LLQLATEGLV KVVPRVGFFV TDVDEKFIRE
4P96  PAERELSELI GVTRTTLREV LQRLARDGWL TIQHGKPTKV NQFMETSGLH
2HS5  S.EPDICAAL DVSRNTVREA FQILIEDRLV AHELNRGVFV RVPTAEDITE
3C7J  R.QQELATLF GVSRMPVREA LRQLEAQSLL RVETHKGAVV APLITEDAVD
4P9F  I.TKNLAEQL GMSITPVREA LLRLVSVNAL SVAPAQAFTV PEVGKRQLDE
3IHU  V.ETDLVAHF GVGRNSVREA LQRLAAEGIV DLQRHRGAVI RRLSLQETLD
1E2X  PAERELSELI GVTRTTLREV LQRLARDGWL TIQHGKPTKV NNFWETSGLN
2DI3  PSERALSETL GVSRSSLREA LRVLEALGTI STATGSGPRS GTIITAAPGQ

   C-terminal domain
    93     α4                          α5                        129
CitO  IYDIRKSLDT LATIKAMELM T.PEDFDELE ALLLEGEQ.. ..........
3FMS  TIETRIMMEV FCLENYFDKI AGSEELLEIK GEIDDVEK.. ..........
4P96  ILDTLMTLDA ENATSIVEDL LAARTNISPI FMRYAFKLNK ESAERIMINV
2HS5  LYICRRVVEC AGVNGFDPAT G...DLSRVA EALDLADE.. ..........
3C7J  AYALRILLES EALRLSIPLL D.ADDLAAAK GYIEQLEV.. ..........
4P9F  INRIRYELEL MAVALAVENL T.PQDLAELQ ELLEKLQQ.. ..........
3IHU  VLDVAERMTG LLARAATRGS GNQPQVQALR ASVQALVA.. ..........
1E2X  ILETLARLDH ESVPQLIDNL LSVRTNISTI FIRTAFRQHP ..........
2DI3  ALSLSVTLQL VTNQVGHHDI YETRQLLEGW AALHSSAERG D.........

   130                         α5                        α6  140
CitO  .......... .......... .......... .......... YNKNNQVDDL
3FMS  .......... .......... .......... .......... SAKREIFDDS
4P96  IESCEALVNA PSWDAFIAAS PYAEKIQQHV KEDSEKDELK RQEILIAKTF
2HS5  .......... .......... .......... .......... RYAVEDWTGV
3C7J  .......... .......... .......... .......... ETDFGQIGRL
4P9F  .......... .......... .......... .......... AQEKGDMEQI
3IHU  .......... .......... .......... .......... AEKAQDGETF
1E2X  .......... .......... .......... DKAQEVLATA NEVADHADAF
2DI3  .......... .......... .........W DVAEALLEKM DDPSLPLEDF

   141       α6                   α7                   α8  186
CitO  LQNFSDFNSF IYTKSQM... LRLKKIVTDL HAYLIYFRDI AIRASERRSI
3FMS  DER...IHKL FIRASGN... ELIISLYEKI WDRIDLVRHL NERYVVSN..
4P96  NFYDYMLFQR LAFHSGNQIY GLIFNGLKKL YDRVGSYYFS NPQARELAME
2HS5  GTADIHFHSA LASLNNS... NRIDELMRSV WNEARLVFHV MDDAHRFHGP
3C7J  NR...MFHLS LYAKTHN... KRLMRLVEEG LNEEERFLRF NLSDMGLGKL
4P9F  INVNRLFRLA IYHRSNM... PILCEMIEQL WVRMGPGLHY LYEAINPAEL
3IHU  SNARRHFYRT LLEMGDN... RELRRLFPTI HMPIVHAQHR LASLRQMR..
1E2X  AELDYNIFRG LAFASGNPIY GLILNGMKGL YTRIGRHYFA NPEARSLALG
2DI3  LRFDAEFHVV ISKGAEN... PLISTLMEAL RLSVADHTVA RARALPDWRA

   187       α8                        α9                    231
CitO  ....ALEEHW LIFRGMKTKN IDQITLLTHE HLNRSLQFIL KEMERRQIE.
3FMS  ......REHK ELIERIISGD KEGAIEKLKE HLKNVEAETI KNLYTYERS.
4P96  .......FYR QLLAVCQSGE REHLPQVIRQ YGIASGHIWN QMKMTLPSNF
2HS5  ....YLTRNH EIYDALAAGN TEAAGQLLKT YLEDAEAQIL GAYRPVSG..
3C7J  ....SQDDHW QLLRLAEQKA IEPCVEALQY HLNRGVQAVT QYLNSKKATK
4P9F  R..EHIENYH LLLAALKAKD KEGCRHCLAE IMQQNIAILY QQYNR.....
3IHU  .....LDDYR RIATAVLAGE PDAAEAAGAA HVKNVRGAIL DRQPA.....
1E2X  .......FYH KLSALCSEGA HDQVYETVRR YGHESGEIWH RMQKNLPGDL
2DI3  TSARLQKEHR AILAALRAGE STVAATLIKE HIEGYYEETA AAEA......

CitO  .......... ..
3FMS  .......... ..
4P96  TEDDC..... ..
2HS5  .......... ..
3C7J  AKSTRAIKKN PA
4P9F  .......... ..
3IHU  .......... ..
1E2X  AIQGR..... ..
2DI3  .......... ..
```

Supplementary Figure 1. Multiple sequence alignment of CitO and its structural homologs. Proteins from *Enterococcus faecalis* CitO (EFT42061, GI:315030129); *Thermotoga maritime* TM0439 (PDB: 3FMS, GI:223365908); *Vibrio cholerae* FadR (PDB: 4P96, GI:753535939); *Rhodococcus* Sp. (PDB: 2HS5, GI:114794493); *Pseudomonas Syringae* Pv. Tomato Str. Dc3000 PS5454 (PDB: 3C7J, GI:168988953); *Escherichia coli* McbR (PDB: 4P9F, GI:747155233); *Xanthomonas Campestris* Pv. Campestris Str. ATCC 33913 Xcc3681 (PDB: 3HIU, GI:254221083); *E. coli* FadR (PDB: 1E2X, GI:12084765); and *Corynebacterium glutamicum* LldR (PDB: 2DI3, GI:145579153). Residues mutated in this work and deduced to be involved in citrate binding within CitO are highlighted in bold. The important residues for metal binding are boxed. Mutated residues in CitO are highlighted in black, whereas the conservation of these residues is shown in gray. The amino acid numbers are relative to those in CitO. The predicted secondary structure elements are showed on top of the alignment, α helices as rectangles and β barrels as arrows.

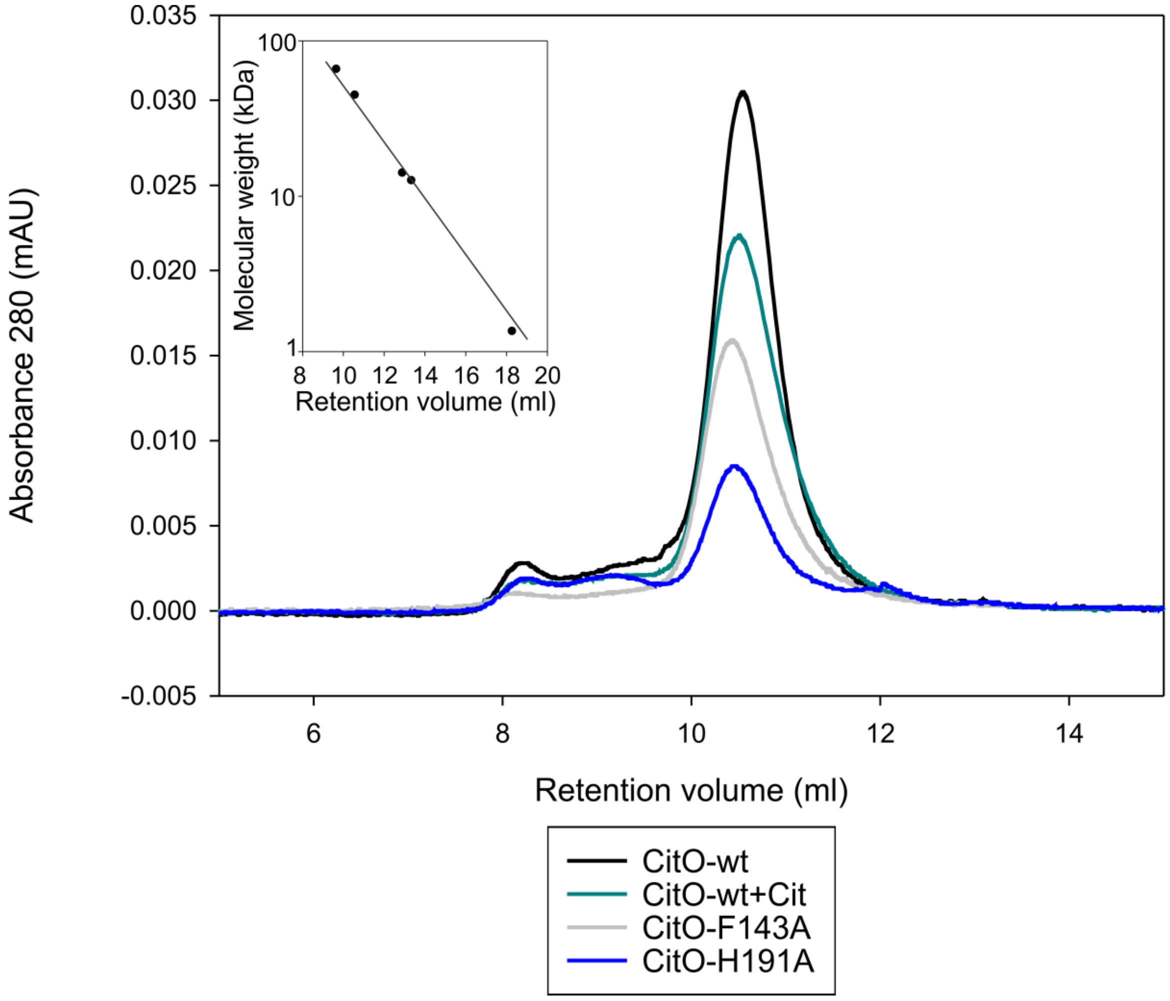

Supplementary Figure 2. Size exclusion chromatography analyses of CitO and its mutant variants. CitO-wt (black line), CitO-wt preincubated with citrate (dark cyan line), CitO-F143A (gray line) or CitO-H191A (blue line) were injected into a prepacked Superdex 75 column as described under "Materials and Methods" The inset shows the calibration curve. The standards used were Bovine serum albumin (66 kDa), Albumin (45 kDa), Lactalbumin (14.2 kDa), Cytochrome C (12.7 kDa), and Vitamin B12 (1.36 kDa). mAU, milliabsorbance units.
